# Adaptive differentiation of *Festuca rubra* along a climate gradient revealed by molecular markers and quantitative traits

**DOI:** 10.1371/journal.pone.0194670

**Published:** 2018-04-04

**Authors:** Bojana Stojanova, Mária Šurinová, Jaroslav Klápště, Veronika Koláříková, Věroslava Hadincová, Zuzana Münzbergová

**Affiliations:** 1 Department of Biology and Ecology, Faculty of Science, University of Ostrava, Ostrava, Czech Republic; 2 Department of Botany, Faculty of Science, Charles University, Prague, Czech Republic; 3 Institute of Botany, Academy of Sciences of the Czech Republic, Průhonice, Czech Republic; 4 Scion (New Zealand Forest Research Institute Ltd.), Whakarewarewa, Rotorua, New Zealand; National Cheng Kung University, TAIWAN

## Abstract

Species response to climate change is influenced by predictable (selective) and unpredictable (random) evolutionary processes. To understand how climate change will affect present-day species, it is necessary to assess their adaptive potential and distinguish it from the effects of random processes. This will allow predicting how different genotypes will respond to forecasted environmental change. Space for time substitution experiments are an elegant way to test the response of present day populations to climate variation in real time. Here we assess neutral and putatively adaptive variation in 11 populations of *Festuca rubra* situated along crossed gradients of temperature and moisture using molecular markers and phenotypic measurements, respectively. By comparing population differentiation in putatively neutral molecular markers and phenotypic traits (*Q*_*ST*_-*F*_*ST*_ comparisons), we show the existence of adaptive differentiation in phenotypic traits and their plasticity across the climatic gradient. The observed patterns of differentiation are due to the high genotypic and phenotypic differentiation of the populations from the coldest (and wettest) environment. Finally, we observe statistically significant covariation between markers and phenotypic traits, which is likely caused by isolation by adaptation. These results contribute to a better understanding of the current adaptation and evolutionary potential to face climate change of a widespread species. They can also be extrapolated to understand how the studied populations will adjust to upcoming climate change without going through the lengthy process of phenotyping.

## Introduction

Understanding how climate change will affect the evolution of existing populations is one of the greatest challenges of evolutionary ecology at present. Climate change will result in novel environments but also increased environmental variation, both of which will generate new selective pressures [1]. Existing populations will have to adjust to these environmental changes in order to avoid extinction. Predictions of the adaptive response of existing populations to these new environmental conditions have practical application in conservation biology or management of invasive species, but will also help us to understand the fundamental evolutionary and ecological mechanisms of rapid adjustments to environmental variation [2,3].

Three mechanisms are commonly distinguished as possible responses to climate change–migration, genetic adaptation and adaptive plasticity [3–5]. In plants, it has been shown that migration is limited–for many plant species, the migration front does not allow to keep up with the pace of predicted local climate change [6]. Genetic adaptation is the result of selection acting on a population or taxon causing heritable modification of the trait(s) under selection, which will, in turn, increase individual fitness [7]. If a population has sufficient genetic variation upon which selection can act, adaptation can occur on a relatively small time scale [3,8]. Organisms can also respond to climate change through phenotypic plasticity–the ability of a given genotype to modify its phenotype in response to environmental variation. If a plastic genotype has higher fitness across variable environments than a non-plastic one, then plasticity is adaptive [9,10]. Adaptive plasticity can itself be the subject of selection [11,12] and differ between populations [13].

When the existing populations encounter novel climates, the new selective pressures to which they will have to adapt will result in population divergence from their current structure [14]. Understanding and quantifying this divergence is thus a necessary step towards understanding the effects of future climate change on the adaptive evolution of present populations. One simple way to do this is to study the adaptive differentiation of existing populations that are exposed to a climatic gradient, i.e. space for time substitutions [15]. By replacing temporal variation with spatial variation, it is possible to study long-term processes, such as population evolutionary response to climate change, without relying on predictive simulations and selection experiments and when long-term data is not available [16–18]. However, populations can also differentiate in the absence of divergent selection, because of random evolutionary processes such as genetic drift [19]. To study the adaptive response of populations, it is thus necessary to separate population differentiation due to selective and random evolutionary processes. A common method to do this is to compare population differentiation based on quantitative genetic or phenotypic traits (*Q*_*ST*_) with that based on putatively neutral molecular markers (*F*_*ST*_) [20–24]. *F*_*ST*_ is the neutral baseline against which population differentiation is measured. If *Q*_*ST*_ = *F*_*ST*_, then the observed differentiation in phenotypic traits is solely due to random evolutionary processes, whereas if *Q*_*ST*_ > *F*_*ST*_, then the phenotypic traits have likely differentiated under the effects of divergent selection, which were higher than random evolutionary processes.

In this study, we estimated population differentiation along a temperature and precipitation gradient of eleven populations of *F*. *rubra* for phenotypic traits and their plasticity, which are putatively under selection, and for molecular markers, which are supposedly not under selection. Our model species is a perennial grass, *Festuca rubra*, which has shown significant differentiation in traits and their plasticity on various spatial scales [25–27]. We used plants originating from locations of different temperature and precipitation from a unique natural grassland ‘climate grid’ established in western Norway [22,28]. We examined the relationship between the phenotypic, molecular and environmental data, with the goals to i) detect patterns of adaptive differentiation among the studied populations, ii) test whether these patterns are consistent with adaptation to climate change. If patterns of differentiation are detected, regardless of their causes, we further iii) tested the relationship between neutral molecular markers and phenotypic traits. Based on the space for time substitution approach, these results will provide input about the capacity of *F*. *rubra* in western Norway to sustain future climate change.

## Materials and methods

### Studied species

*Festuca rubra* L. is a common perennial grass species of temperate grasslands in Europe. *F*. *rubra* ssp. *rubra* used in the experiment is a widespread hexaploid type from the *F*. *rubra* complex. It reproduces sexually through outcrossing [29] as well as vegetatively by producing both intravaginal and extravaginal tillers on rhizomes. Clones of *F*. *rubra* possess considerable genetic variability and plasticity [25–27].

### Study sites and sampling

The study material was sampled in a natural climate grid in Western Norway (the SeedClim project, [28,30]). The 12 natural grassland sites represented a full cross design of four levels of mean annual precipitation (noted 1–4 in increasing order, S1 Fig) and three levels of summer temperature (ALP—alpine, SUB—subalpine and BOR—boreal, in increasing order, S1 Fig). Sites were selected specifically to keep management and history, bedrock, slope, aspect and vegetation types as constant as possible [28,30]. The geographical distance between sites ranged from 0.65 to 175 km (average 15km, S1 Fig). The sampling areas were of variable size, depending on *F*. *rubra* density, plot morphology and vegetation homogeneity (S1 Table). Transects were established, and plants were collected until the required number of plants was reached. To avoid sampling clones of the same genotype, the minimum distance between two sampled plants was 1 m. In June 2014, 25 plants were collected at each locality and transported to the experimental garden of the Institute of Botany, Academy of Sciences of the Czech Republic in Průhonice, Czech Republic (49°59'38.972"N, 14°33'57.637"E; mean annual temperature 8.6°C and regular watering during the vegetation season) and planted into pots. The hexaploid status, which is the most widespread cytotype of *F*. *rubra*, was confirmed using flow cytometry using the protocol described in [31]. In locality ALP2, no *F*. *rubra* hexaploids occurred, so this site was excluded from further analyses. To ensure that each of the collected plants corresponded to a single genotype, plants were reduced to a single ramet at the end of June 2014. The maternal ramets were grown in a greenhouse for nine months to acclimate them to growth in pots and eliminate potential differences due to growth in different environments of origin.

### Climatic chamber experiments

In March 2015, four single young ramets originating from the maternal ramets were taken from each plant, and each was placed in a different growth chamber. Each growth chamber simulated the spring and summer conditions in one of the four most extreme natural environments observed in the climate grid (ALP1, ALP4, BOR1, BOR4). The temperature in the growth chamber differed between the cold and warm treatments and changed over the growing season following the course of temperature at the natural localities (Table 1). Soil moisture was monitored continuously during the whole experiment and watering was modified to ensure constant moisture throughout the experiment. In brief, the dry regime plants were watered with about 20 mL of tap water per plant applied to the trays if the soil moisture was lower than 15%. In the wet regime, plants were cultivated under full soil saturation with about 1.5 cm of water in the bottom of the tray. For all the regimes, the same day length and radiation were used, i.e. 16 hours of full light (6 am– 10 pm) and 4 hours of full dark with a gradual change of light availability in the transition between the light and dark period over 2 hours. For an illustration of the experiment see S3 Fig. For a detailed description of the experimental protocol and the growth chamber conditions see [27].

**Table 1:** Climatic chamber settings throughout the study. Reproduced with modification from [27].

	Alpine			Boreal		
Day	Min (°C)	Max (°C)	Av (°C)	Min (°C)	Max (°C)	Av (°C)
1–4	5	15	9.8	5	16	10.1
5–25	3	12.5	7.5	3	16	9.2
26–46	3	12.5	7.5	3	18.5	10.2
46–67	3	12.5	7.5	3	24.3	12.5
68–88	3	14.5	8.4	3.4	25	12.9
89–176	3	14.7	8.5	5	23.8	14.8

The temperatures in the growth chambers were set to reproduce daily (minimal and maximal) and seasonal temperature variation during the spring in the alpine and boreal localities.

### Traits measurement

At the end of August 2015 (21 weeks after placement in the growth chamber) we assessed photosynthetic activity, water potential and stomatal density and size as physiological traits. Photosynthetic activity was assessed through the measurements of the maximum quantum yield of primary PS II and the performance index for energy conservation from photons absorbed by PS II antenna [32]. These traits were measured using FluorPen FP-100 MAX/USB (Photon System Instruments, Brno, Czech Republic). Measurements were made in the dark, at 25°C, at the same time of the day for all plants (9.00–10.30pm). Two hours prior to the measurements plants were kept in the dark by switching off the growth chamber lights. Three measurements were made per plant, and their average was used in further analyses. Stomatal density and size were measured at the end of the experiment, prior to the harvest, using imprints that were observed with an Olympus BX53 microscope with Nikon DS-Fi2 camera for live projection and LIM NIS Elements Ar 4.11 software. Stomatal density was assessed in a 500μm square edge grid in three randomly chosen positions on one leaf. For each of the selected positions, the length of three randomly chosen stomata was measured and then averaged to obtain stomatal size. To measure water potential, all plants were saturated with water one day prior to their harvest. After harvest, about five leaves (or less depending on the volume they occupied) were placed in 1-mL Braun Omnifix-F syringes with a seal from synthetic rubber (B. Braun Melsungen AG, Melsungen, Germany) and frozen to break down the cell walls. After the material was unfrozen, cell cytoplasm was extracted by applying pressure on the plant sample with the syringe. Water potential measurement was made on 10μl of cytoplasm placed on 80 g/m2, wood pulp filter paper, using dew point micro voltmeter Wescor HR33 (Wescor Inc., Logan, USA).

At the end of the experiment, the number of intravaginal and extravaginal ramets and the plant height were assessed. The total number of ramets was calculated as the sum of intravaginal and extravaginal ramets. Finally, the plants were harvested, dried at 60°C to constant weight, and the aboveground (cut at the 3 cm height above the soil surface), and belowground (rhizome and root separately) biomasses were weighed. We calculated the below:aboveground biomass ratio.

We calculated the plasticity of all of the measured traits within each genotype as (|trait_MAX_−trait_min_|)/trait_MAX_ where trait_MAX_ and trait_MIN_ were respectively the maximal and minimal value of the trait measured on the same genotype across the four growth chambers [33]. We chose this index as it is easy to use, robust, widely applied approach that allows comparison among traits [34]. This estimate only characterized the maximal plastic capacity of an individual in variable environments without taking into account the direction of the plastic response or the change in intensity with environmental variation (i.e. reaction norms).

### Microsatellite data

All of the studied genotypes were genotyped using standard DNA extraction and amplification methods for the purpose of a different study. For the genotyping we used extra ramets of the clones used in the growth chamber experiment. In short, total genomic DNA was extracted from dry leaves stored in silica gel using a silica-based DNeasy Plant Mini kit (QIAGEN, Dusseldorf, Germany). Quality and quantity of extracted DNA were measured by NanoDrop 2000 (Thermo Scientific, Waltham, USA), and all the samples were normalized to 20 ng/μl for subsequent PCR. Four microsatellite loci were amplified using *Festuc*a and *Festuca—Lolium* complex specific primers HVM20 and HVM3 [35] and B4-D9 and B3-B8 [36]. Multiplex PCR reaction was designed combining all microsatellite primers to one reaction. Multiplex PCR reaction contained 2.5 μl QIAGEN Multiplex PCR Master Mix (Qiagen, Hilden, Germany), 0.25 μl of HVM 20 primers, 0.05 μl of HVM3, B3-B8, B4-D9 primers (10 μM each in initial volume) 0.7 μl H2O and 20 ng of DNA dissolved in 1 μl TE buffer. An initial denaturation step at 95°C for 15 min was followed by 40 cycles of denaturation (95°C for 30 s), annealing (60°C for 90 s) and extension (72°C for 60 s) steps, and a final extension step at 72°C for 10 min. A 1 μl aliquot of the PCR product was mixed with 11 μl of a 120: 1 solution of formamide and size standard GeneScan 500 LIZ (Life Technologies, Foster City, CA, USA). Fragment lengths were determined by capillary gel electrophoresis with an ABI 3130 Genetic Analyzer using Gene Mapper 4.0 (Life Technologies) and the peaks were scored manually. Overall, the four loci produced 62 alleles (20, 16, 15 and 11 for loci B3-B8, HVM2, B4-D9 and HVM3 respectively, S5 Table).

### Data analysis

All analyses were done in R 3.2.5 [37]. The effect of initial ramet size (i.e. at the beginning of the growth chamber experiment) on phenotypic trait values and their plasticity was non-significant when tested in generalized linear models as we attempted to standardize it when setting up the experiment [27]. Therefore we did not include initial ramet size as an explanatory variable in our analyses.

#### *Q*_*ST*_*-F*_*ST*_ and *P*_*ST*_*-F*_*ST*_ comparisons

To estimate the population quantitative genetic differentiation (*Q*_*ST*_), we first estimated additive genetic variance and between population variance following a similar approach as in [38] using the animal model [39]. Within and between population quantitative genetic variation were estimated using REML approach in ASReml-R[40] as follows: *y =* X*β +* Z*p* + Z*a* + *ε* where X and Z–incidence matrices assigning fixed and random effects to measurements in vector *y*, *β* –the vector of the fixed effects of growth chamber climate and any other unspecific chamber effects, *p*~N(0,*I*σ_p_^**2**^)–the vector of random population effects, with *σ*_*p*_^*2*^ –between population variance and *I* is identity matrix, a~N(0, 2*G*σ_a_^**2**^)–the vector of random individual plant genetic effects, with *σ*_*a*_^*2*^—within population (additive genetic) variance and *G*–Loisselle’s kinship coefficient [41] matrix estimated in SPAGeDi 1.5 [42] using the four microsatellite markers of this study (S5 Table). Since trait plasticity was calculated as the difference of trait values measured for a single genotype grown in different growth chambers, within and between population quantitative genetic variation of trait plasticity was estimated with the model *y =* X*β* + Z*p* + Z*a* + *ε* Narrow-sense *Q*_*ST*_ was then calculated for trait values and plasticity as $Q_{ST}=\frac{{\hat{\sigma }}_{p}^{2}}{{\hat{\sigma }}_{p}^{2}+{\hat{\sigma }}_{a}^{2}}$ with ${\hat{\sigma }}_{p}^{2}$ and ${\hat{\sigma }}_{a}^{2}$ being the REML estimates of between and within-population genetic variance.

Kinship coefficient estimates based on four loci could be imprecise and downwardly bias the estimates of within-population variance, resulting in an overestimate of *Q*_*ST*_. We thus estimated the lower bound of *Q*_*ST*_ through the population phenotypic differentiation index (*P*_*ST*_) as defined in [43]. Within and between population variation were estimated using a general mixed model as follows: *y = β + b + ε*, where *β* –the vector of the fixed effects of growth chamber and any other unspecific chamber effects, *b*~N(0,*σ*_*b*_^*2*^)–the vector of random population effects, with *σ*_*b*_^*2*^ the variance of random population effects, and *ε~N(0*, *σ*_*w*_^*2*^)–the vector of residuals, with *σ*_*w*_^*2*^ the residual population variance. We then calculated *P*_*ST*_ for trait values and plasticity as $P_{ST}=\frac{\frac{c}{h^{2}}{\hat{\sigma }}_{b}^{2}}{\frac{\hat{c}}{h^{2}}\sigma_{b}^{2}+{\hat{\sigma }}_{w}^{2}}$ with ${\hat{\;\sigma }}_{b}^{2}$ and ${\hat{\sigma }}_{w}^{2}$ being the estimates of within and between population variation, *c* the proportion of between population variance that is due to additive genetic factors only, and *h*^*2*^ trait heritability, or the proportion of within-population genetic variance that is due to additive genetic factors. We assume *c = h*^*2*^ *= 1*, meaning that all of the observed phenotypic variance is due to additive genetic factors only [44]. The assumption *c = 1* plausible in our study, given that all populations are grown in a common environment, which reduces between population differences that can be due to environmental factors. Assuming *h*^*2*^ *= 1* is clearly an overestimate of the additive genetic component of *σ*_*w*_^*2*^, but it is relevant in our case because we want to estimate the lower boundary of *P*_*ST*_, which is done by maximising the within-population variance.

To test for the effects of selection on population divergence in quantitative traits, we used the method described in [45] as implemented in an R script available from [46]. In brief, a neutral *Q*_*ST*_*-F*_*ST*_ (*P*_*ST*_*-F*_*ST*_) distribution was simulated using the Lewontin-Krakauer distribution [47], and the estimates of *F*_*ST*_ (from molecular markers), and of ${\hat{\sigma }}_{p}^{2}$ and ${\hat{\sigma }}_{a}^{2}$ for *Q*_*ST*_ (of ${\hat{\sigma }}_{b}^{2}$ and ${\hat{\sigma }}_{w}^{2}$ for *P*_*ST*_). The quantile of the observed ${\hat{Q}}_{ST}-{\hat{F}}_{ST}\left({\hat{P}}_{ST}-{\hat{F}}_{ST}\right)$ value compared against the neutral distribution was obtained in order to determine the p-value of the null hypothesis that *Q*_*ST*_ (*P*_*ST*_) equals *F*_*ST*_. This method was particularly suitable for our data set, as it gives reliable results when used with relatively few neutral molecular markers, when population differentiation in molecular markers is low, and when the number of populations is relatively high (ten or higher). Given that microsatellite data can have mutation rates that are higher than migration rates, microsatellite based *F*_*ST*_ can be downwardly biased. To avoid this, we also made *Q*_*ST*_*—R*_*ST*_ comparisons, with *R*_*ST*_ being an *F*_*ST*_ analogue based on allele size, calculated following [48]. *R*_*ST*_ should not be affected by the microsatellite mutation rate, provided that microsatellite size variance is proportional to their genetic distance [49,50].

#### Coinertia analysis

Coinertia analysis is a multivariate ordination method that measures the concordance between two data sets. The goal of a COA is to find a multidimensional projection of the two data sets which is a compromise between the maximal variance of each data set and the maximal covariance between the two data sets [51,52]. COA can be used to explore the shared structure between genetic diversity as estimated by molecular markers and phenotypic traits [53]. We used COA analyses to simultaneously examine the relationship between differentiation of molecular markers, phenotypic traits (or plasticity). If this relationship was significant, we further tested how the covariation between traits and markers was affected by environmental variation, instead of proceeding by pairwise comparisons between the three data sets. A significant effect of the environment would confirm that the observed joint population differentiation in traits and markers can be at least partly predicted by the environment, and thus the molecular markers could be used as indicators of the phenotypic value of the individuals of the studied populations.

COA was made using the projections of the first two axes of Principal Component Analysis (PCA) for trait values (or plasticity) and molecular markers. For phenotypic trait values PCA we used the Euclidian distance matrices based on the trait values averaged across growth chambers calculated by the R package ade4 [54], and for molecular markers PCA we used Nei-distances calculated by the R package adegenet [55]. The significance of the correlation between the matrix of phenotypic trait values (or plasticities) and that of neutral genetic patterns was assessed by 999 bootstraps. COA with trait means and individual genetic differentiation will be further referred to as COAmean, and COA with trait plasticity and individual genetic differentiation will be referred to as COAplast.

If significant coinertia was detected between trait values (or plasticities) and molecular markers, we then tested how are the individual projections on the main COA axis (COA axis 1) affected by climate variation. For this, we used ANOVA with temperature and precipitation of origin and their interaction as fixed, quantitative, explanatory variables. Significance levels were estimated using Fisher’s F statistic. A significant ANOVA result meant that the molecular marker variation which was associated with variation of trait value (or plasticity) was also influenced by climate variation. If such a relationship existed, we further tested for alleles that were associates with specific trait values, or which were characteristic for some populations (see below). Note that if allelic frequencies were influenced by phenotypic trait values and/or environmental variables, it does not mean that they were under selection, as microsatellite loci are by default considered to be neutral markers. This association could have been caused by pure genetic drift, or non-random mating patterns. Independent of the causes of the association, the alleles associated with phenotypic traits and climatic variables could further be used to identify individuals with specific phenotypes or thriving in specific climates.

#### Testing for the causes and consequences of population genetic differentiation

We tested for isolation by distance (IBD) and isolation by adaptation (IBA) by estimating the proportion of genetic differentiation between populations explained by geographical and environmental distances, respectively, using Mantel tests (R package vegan). The genetic distance matrix was based on Bruvo distances (R package polysat,[56]), and the geographic and environmental (combining temperature and precipitation data) distances were based on Euclidean distances. Significance testing was made with 999 bootstraps. In addition to testing the effect of geographical and environmental distances on their own, we used partial Mantel test to examine the effect of environmental distance after accounting for geographical structure, and of geographical distances after accounting for environmental structure.

We also tested the relationship between population genetic diversity and population trait means or plasticity using Pearson’s product moment correlations. Since the estimate of genetic diversity in hexaploid organisms is not straightforward because complete information about allelic frequencies cannot be obtained [57,58], several different estimators were used: number of alleles, number of effective alleles, allelic richness and expected heterozygosity and Pons and Petit’s index of population genetic diversity of non-ordered alleles relationship between individual genotypes [59]. All of these estimators were calculated with SPAGeDi 1.5 [42]. Being based on expected rather than observed allele frequencies, these estimators are informative about the population effective size. Thus a significant correlation between genetic diversity and trait means could indicate that populations with a lower effective size are phenotypically highly differentiated, possibly because of the effects of IBD or IBA [60].

#### Testing the association between molecular markers and traits and their plasticities in the studied populations

The statistical significance of the association between specific molecular marker alleles and phenotypic traits was tested with Multivariate Analysis of Variance (MANOVA). As the total number of alleles was higher than degrees of freedom associated with the phenotypic traits, we first selected the alleles that were most suitable for the analyses based on abundance criteria. We thus excluded rare or private alleles by selecting only alleles that were either present in seven or more populations, or alleles that were present in less than seven populations but they had population frequencies higher than 0.05. In locus HVM3 we excluded two alleles whose frequency was equal or close to 1 in all populations. Then we tested the effect of all alleles within each locus separately by using step-wise selection procedure within MANOVA. All alleles that were retained for each locus after the step-wise selection were combined together as explanatory variables in a new MANOVA. To make sure that the order of introduction of the alleles did not affect the MANOVA results, type 3 testing was carried out by permuting the introduction order of the alleles. To enable comparison between traits with different units, trait means were z-transformed using the scale function in R. Significance levels were assessed using the Pillai statistic. When a significant effect of at least one allele was observed, we performed individual ANOVAs for each trait separately in order to extract the coefficient of the slope associated with each of the tested alleles.

## Results

### Patterns of divergent selection

The global population genetic differentiation was very low, regardless of the estimator (*F*_*ST*_: 0.039 ± 0.011 standard error, *R*_*ST*_: 0.057 ± 0.047 standard error). *Q*_*ST*_ estimates were obtained for ten out of the twelve phenotypic trait values. *Q*_*ST*_ values for below:aboveground biomass and water potential could not be estimated with our model because their variance components were fixed at the boundary. *Q*_*ST*_ estimates for trait values were generally low (0.072 ± 0.024). Six trait values showed *Q*_*ST*_*-F*_*ST*_ significantly different from the neutral *Q*_*ST*_*-F*_*ST*_ distribution (Table 2) and could be under divergent selection. Five of these traits were significantly different than the neutral *Q*_*ST*_*-R*_*ST*_ distribution as well (Table 2). *Q*_*ST*_ estimates for plasticity were obtained for nine phenotypic traits. *Q*_*ST*_ estimates for plasticities were generally much higher than *Q*_*ST*_ for trait values (0.477 ± 0.131), and close to 1 for stomatal density, water potential and number of ramets. However, the precision of the *Q*_*ST*_ estimates for trait plasticity was lower than that for trait values (see standard errors in Table 2). This is likely because the sample size for the *Q*_*ST*_ estimates of plasticity is four times smaller than that of *Q*_*ST*_ for trait values. Both *Q*_*ST*_*-F*_*ST*_ and *Q*_*ST*_*-R*_*ST*_ were significantly different from the neutral distribution for plasticity of morphological traits, most of the plasticities of physiological traits, but not for the plasticities of the two resource acquisition traits (Table 2). Thus according to this result plasticity of morphological and physiological traits is likely under divergent selection. The lower bound of *Q*_*ST*_, estimated as population phenotypic differentiation, *P*_*ST*_, did not show these patterns of divergent selection (Table 2)–neither trait values nor plasticity showed patterns consistent with the effects of divergent selection except for the value of stomata density.

**Table 2:** Animal model estimates of population differentiation (*Q*_*ST*_ and *P*_*ST*_) for phenotypic trait values and their plasticity.

	Trait value			Trait plasticity			Trait value				Trait plasticity			
Trait	P_ST_	std. error	PST—F_ST_	P_ST_	std. error	PST—F_ST_	Q_ST_	std. error	QST—F_ST_ p-value	QST—R_ST_ p-value	Q_ST_	std. error	QST—F_ST_ p-value	QST—R_ST_ p-value
			p-value			p-value								
Plant Height	0.045	0.021	0.308	0.027	0.02	0.776	0.097	0.056	**0.007**	0.067	0.592	0.758	**<0.0001**	**<0.0001**
Number of ramets	0.033	0.016	0.617	0.011	0.011	0.979	0.059	0.035	0.117	0.41	NA	NA	NA	NA
% extravaginal ramets	0.029	0.014	0.718	0.009	0.011	0.988	0.072	0.043	**0.044**	0.221	0.019	0.121	0.895	0.953
Aboveground biomass	0.033	0.017	0.598	0.036	0.024	0.534	0.132	0.074	**0.0002**	**0.0101**	0.644	0.766	**<0.0001**	**<0.0001**
Belowground biomass	0.032	0.016	0.642	0.025	0.016	0.836	0.132	0.082	**0.0005**	0.101	0.866	1.618	**<0.0001**	**<0.0001**
Rhizome biomass	0.020	0.011	0.893	<0.0001	<0.0001	0.997	0.048	0.037	0.2398	0.609	<0.0001	<0.0001	**0.997**	**0.994**
Below:aboveground biomass	<0.0001	0.001	0.997	0.018	0.016	0.914	NA	NA	NA	NA	NA	NA	NA	NA
FI.P0	0.009	0.006	0.982	0.007	0.009	0.993	0.014	0.028	0.96	0.974	0.033	0.46	0.67	0.850
Piabs	0.027	0.014	0.756	0.014	0.014	0.960	0.076	0.05	**0.031**	0.1777	0.335	0.69	**<0.0001**	**<0.0001**
Water potential	0.004	0.004	0.993	0.023	0.019	0.824	NA	NA	NA	**NA**	0.988	1.326	**<0.0001**	**<0.0001**
Stomata density	**0.110**	0.049	**0.003**	0.055	0.041	0.203	0.261	0.124	**<0.0001**	**<0.0001**	NA	NA	NA	NA
Stomata size	0.028	0.018	0.731	0.020	0.024	0.951	0.05	0.063	0.223	0.579	0.337	0.804	**<0.0001**	**<0.0001**

p-values were calculated using the Whitlock and Guillaume [45] method for *P*_*ST*_*—F*_*ST*_, *Q*_*ST*_*—F*_*ST*_ and *Q*_*ST*_*—R*_*ST*_ comparisons. Significant values are in bold. PI_ABS_−photosynthetic index (see main text for explanations).

### Effect of environmental variation on quantitative traits and molecular markers

**Fig 1:**
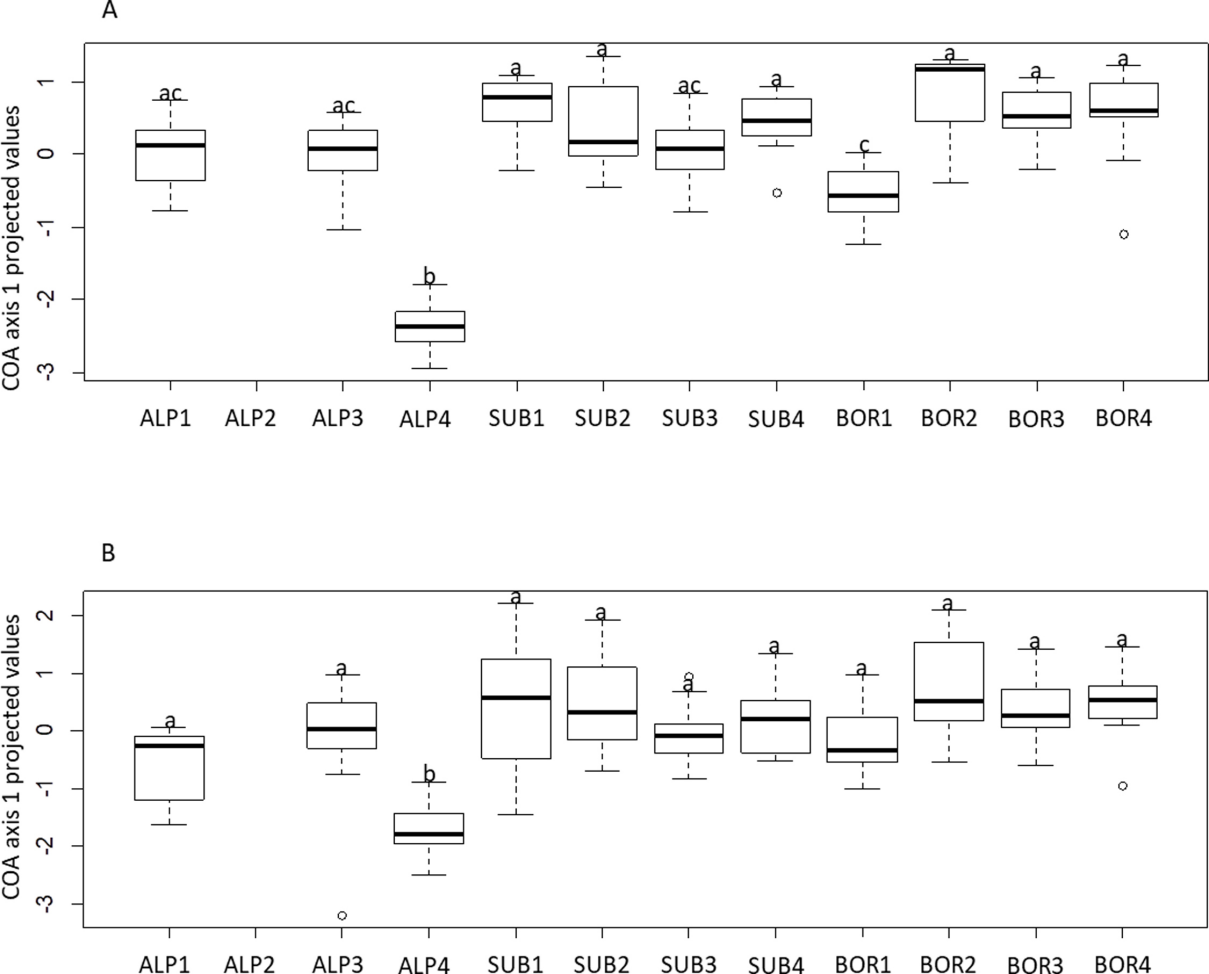
Boxplot of population differentiation along the first COA axis. A. COA between phenotypic trait values and molecular markers B. COA with phenotypic plasticity and molecular markers. Localities with the same letters do not differ significantly according to Tukey’s post-hoc test.

COA results showed significant shared levels of inertia between trait values and molecular markers (COAmean), and between plasticity and molecular markers (COAplast; Table 3). The first axis of COAmean was significantly associated with temperature, precipitation and their interaction. The effect of temperature was due to the differentiation of ALP populations from SUB and BOR populations, whereas the effect of precipitation was not so clear. The interaction effect was mostly due to the differentiation of populations ALP4 and BOR1 whose coinertia patterns were similar to each other and contrasting to the remaining nine populations (Table 3, Figs 1A and S2). The individuals of populations ALP4 and BOR1 were characterised by short leaves, low below- and above-ground biomass; and high below:aboveground biomass ratio, rhizome biomass, proportion of extravaginal ramets, and stomatal density; as well as higher frequencies of the alleles B4-D9 245, B3-B8 281, HVM2 114 and HVM3 159. These qualitative associations between trait values and specific alleles were partly confirmed (and quantified) by the MANOVA results (S2 Table). The first axis of COAplast was affected by temperature and precipitation x temperature, but not precipitation alone (Table 3). Likewise for COAmean, the effect of temperature was due to the differentiation of ALP populations from SUB and BOR populations. The interaction effect was due to the high differentiation of population ALP4 from the remaining ten populations (Table 3, Fig 1B). ALP4 individuals had high plasticity of four growth traits (number of ramets, plant height, aboveground biomass and below ground biomass), and stomata size; low plasticity of resource acquisition and photosynthetic traits; and were associated with the alleles HVM3 117, B4-D9 249, 239 and B3-B8 281 (S2 Fig). These associations were not supported by the MANOVA results. Locality showed a significant effect on the individual projections of COAmean and COAplast (Table 3). The variation explained by locality itself was much higher than by temperature and precipitation combined, suggesting that other factors than climate might contribute to population differentiation.

**Table 3:** Results of coinertia analysis between phenotypic trait values and molecular markers (COAmean) or phenotypic plasticity and molecular markers (COAplast).

Analysis	R	p-value	Variable	Df	F	p-value	explained variation (%)
COAmean	**0.207**	**0.001**	Effect of environment				
			Temperature	**1**	**32.8**	**< 0.001**	**18.16**
			Precipitation	**1**	**5.546**	**0.02**	**3.07**
			Temperature x Precipitation	**1**	**45.28**	**< 0.001**	**25.07**
			Effect of locality				
			Locality	**10**	**28.35**	**< 0.001**	**75.92**
COAplast	**0.193**	**0.001**	Effect of environment				
			Temperature	**1**	**29.49**	**< 0.001**	**20.29**
			Precipitation	1	1.343	0.249	0.92
			Temperature x Precipitation	**1**	**9.446**	**0.003**	**6.50**
			Effect of locality				
			Locality	**10**	**7.886**	**< 0.001**	**44.59**

R–correlation coefficient of the coinertia analysis. p-value was obtained by 999 bootstraps. Significant values are shown in bold. The right part of the table shows the results of ANOVA testing the effect of climate variation (temperature and precipitation) or the effect of locality, on the projected values of the principal COA axis. DF–degrees of freedom, F–Fisher’s F. Significant values are in bold.

### Causes and consequences of population genetic differentiation

According to the Mantel tests, there was no significant correlation between genetic and geographic distances between populations (r = 0.010, p-value = 0.255), even in partial tests taking into account environmental distances (r = -0.005, p-value = 0.471). Environmental distances showed significant correlation with genetic distances when tested alone (r = 0.143, p-value ≤ 0.001) or in partial tests with geographic distances (r = 0.143, p-value ≤ 0.001).

All estimators of population genetic diversity (number of alleles, number of effective alleles, allelic richness and expected heterozygosity) showed similar estimates, thus only the correlations with Pons and Petit’s index of population genetic diversity of non ordered alleles are shown (Table 4). The correlation between population genetic diversity and trait means per population was significant for four traits. Proportion of extravaginal ramets and stomatal density were negatively correlated with genetic diversity, and below- and above-ground biomasses were positively correlated with genetic diversity. In line with this, these two pairs of traits show positive correlations within pair and negative correlations between pairs according to the COA (S2 Fig). Regarding mean trait plasticity, population genetic diversity was significantly and negatively correlated with the plasticity of number of ramets and aboveground biomass, and marginally and positively correlated with proportion of extravaginal ramets (Table 4).

**Table 4:** Results of the Pearson’s product moment correlation of mean population genetic diversity and trait values or phenotypic plasticity mean per population.

	Trait value		Plasticity	
Trait	r	p-value	r	p-value
Plant height	0.324	0.330	-0.196	0.563
Number of ramets	-0.263	0.434	**-0.628**	**0.039**
% extravaginal ramets	**-0.691**	**0.019**	0.593	0.054
Rhizome biomass	-0.263	0.435	-0.348	0.294
Belowground biomass	**0.627**	**0.039**	-0.141	0.680
Aboveground biomass	**0.828**	**0.002**	**-0.613**	**0.045**
Below:aboveground biomass	-0.521	0.100	-0.570	0.067
PI.abs	0.304	0.364	-0.538	0.088
Water potential	0.599	0.052	-0.318	0.341
Chlorophyll fluorescence	0.381	0.248	-0.211	0.533
Stomatal density	**-0.682**	**0.021**	0.234	0.489
Stomatal size	0.098	0.774	-0.513	0.106

## Discussion

### Population differentiation and patterns of selection

Overall, the population genetic differentiation estimated from molecular markers was very low (both *F*_*ST*_ *and R*_*ST*_). These results need to be taken with precaution, as population genetic differentiation in this study can be somewhat underestimated. First, the of lack of exact allele frequency estimates due to the hexaploid status of *F*. *rubra* [57,58] likely overestimates the rare allele frequencies and underestimates the abundant allele frequencies, resulting in an overall underestimate of population differentiation. Second, microsatellite markers are known to have a high mutation rate, which can further underestimate population differentiation [49]. We partly circumvented this second bias using an estimate of population differentiation that takes into account the marker mutation rate, *R*_*ST*_ [48], under the assumption that the microsatellite markers of this study comply to a stepwise mutation model [61]. In spite of these potential biases, testing for isolation by distance in the studied populations using Mantel tests did not show significant structure either, which is consistent with low differentiation of *F*. *rubra* populations due to neutral genetic processes.

Population phenotypic differentiation (*P*_*ST*_) was overall very low, and did not differ significantly from *F*_*ST*_, except for stomatal density. Stomatal density is a trait regulates carbon uptake and water use efficiency of the plant, and as such is highly responsive to environmental variation [62]. Depending on the species, stomatal density response to environmental variation can be highly adaptive [63], and has often been used as an indicator for climate change based on plant fossil records [64,65]. It is thus not surprising, given the overall adaptive response of this trait across multiple species, that even with the conservative *P*_*ST*_*−F*_*ST*_ test, stomatal density shows patterns of adaptive differentiation in our study. For all the other traits, based on *P*_*ST*_*−F*_*ST*_ comparisons, if the selection is acting on these populations, its effects cannot be disentangled from the effects of genetic drift. This result is contradictory to the findings of previous studies using the same approach to estimate the effects of divergent selection [44,66,67] but see [68,69]. *P*_*ST*_ estimates in the previous studies were made on traits measured in natural populations. Thus the observed variation between and within populations could include environmental effects, genotype x environment interactions, and non-additive genetic effects, which can inflate the estimated variances [70]. Since the populations in our study were grown in a common environment, most of the environmental effects on variance estimates were likely cancelled out, resulting in a decreased estimate of between population variance. The effects of the environment were further minimized by growing the maternal ramets in a common garden for nine months prior to the setting of the experiment. This was important as the conditions experienced by the maternal ramets were shown to have strong effects on offspring ramet performance in the same model system [71]. Within-population phenotypic variance, however, comprised non-additive genetic effects, which may increase its value (S3 Table), and result in an overall underestimate of *P*_*ST*_.

Within-population variance, as calculated for *Q*_*ST*_, was unsurprisingly lower than within-population variance as calculated for *P*_*ST*_. This is expected, as for the *Q*_*ST*_ estimates, within-population variance was partitioned into additive variance, explained by the genetic relatedness between individuals, and residual variance, and only the additive variance was used to calculate *Q*_*ST*_. Thus *Q*_*ST*_ estimates obtained with this approach were overall higher than their respective *P*_*ST*_. Furthermore, the majority of trait values and plasticities *Q*_*ST*_ were significantly higher than *F*_*ST*_, consistent with the effects of divergent selection [72,73]. These results are nevertheless to be taken with precaution, as the *Q*_*ST*_ estimates could be inflated. Indeed, the kinship estimates from only four loci could be imprecise, leading to increased “noise” underestimates of the within-population additive variance, σ_a_^2^.

Based solely on the range of *P*_*ST*_ and *Q*_*ST*_ estimates, we cannot reliably conclude regarding the adaptation of the populations in this study to temperature and precipitation variation. However, the results of coinertia analyses show a significant effect of temperature and precipitation on the shared inertia. Furthermore, a previous study, equivalent to a ‘reciprocal climate common garden experiment’, using the same plant material, showed that the climate of origin has a small, but significant effect on phenotypic trait values [27]. The intensity of the adaptive response is rather low, as suggested by the weak effects of selection observed in these populations (estimated between -0.04 and 0.132, Stojanova et al. pers. obs.), which could explain the absence of clear cut patterns of adaptive population differentiation in our study. Taken altogether, these results cannot quantify the adaptive response of *F*. *rubra* populations in western Norway, but they can be considered as qualitative indicators of its existence.

### Relationship between molecular markers, quantitative traits and environmental variation

The observed population differentiation in our study this seems to be mainly because populations from the coldest, alpine, climate are highly differentiated from populations from subalpine and boreal climate. Indeed, COA results show that alpine populations, and in particular the one from the locality with the highest precipitation (ALP4), have more effective foraging abilities (high proportion of extravaginal ramets and rhizome biomass) and slower growth (low number of ramets, leaf length, biomass measures). Increased foraging and reduced size is a pattern commonly observed in plants in response to different types of unfavourable environments [74–76]. In line with this, foraging and growth traits both show significant, but opposing correlations of their population means with population genetic diversity. This is probably due to the fact that alpine populations, notably ALP4, have reduced effective population size (indicated by their lower genetic diversity, S4 Table), while at the same time exhibiting adaptive trait combinations to their climate of origin. The causes of this association are discussed in the following section.

Interestingly, our results suggest that trait plasticity could also be under divergent selection. In our study, differentiation in plasticity is solely due to the higher plasticity of alpine populations according to the coinertia analyses, although alpine and boreal populations are facing the same absolute environmental distances (with opposing signs) in the growth chambers. This could be because alpine populations need to efficiently use the short ‘windows of opportunity’ when favourable conditions occur in the harsh alpine environment to rapidly increase their growth, and have thus evolved higher plasticity to cope with local, micro-environmental variations [27]. This hypothesis assumes adaptive phenotypic plasticity, i.e. that plastic individuals would have higher fitness than non-plastic ones in the studied environments [9,10]. If plasticity is non-adaptive for the studied populations, then the high phenotypic variability in alpine populations can also be due to the lack of canalisation [77]. In this case, the inability of the plants originating from extreme climatic environments to produce a stable phenotype when facing environmental variation results in non-adaptive plasticity. Non-adaptive phenotypic plasticity has indeed been observed in alpine plant populations (e.g. [70]). However, to evaluate the (non) adaptive character of plasticity in *F*. *rubra*, it is necessary to relate the degree of plasticity of each individual to an estimate of its fitness [9,10,78], which is a complicated task in clonal grasses. Indeed, the high longevity of clonal ramets (up to several hundred years, [79]) makes the lifetime fitness related to clonal reproduction nearly impossible to assess. In addition, our experimental plants flowered very rarely, and early flowering in long-lived perennials may not be an indication of high fitness, but a response to stress [80].

The plasticity *Q*_*ST*_ estimates have rather low precision and non-significant lower bound estimates (*P*_*ST*_), and thus need to be considered with precaution. Only a few other studies have tested for among-population differentiation in phenotypic plasticity using *Q*_*ST*_*-F*_*ST*_ comparisons and have found low or non-significant *Q*_*ST*_ for plasticity [46,81–83]. However, the result found in our study is likely due to the reduced sample size available for the estimates, rather than the absence of a population differentiation mechanism. Indeed, it has been shown that plasticity is an evolving trait like any other quantitative trait [84], and can respond to selection imposed by controlled climatic variation [13].

COA results showed significant positive covariation between molecular markers and phenotypic trait values; and between molecular markers and phenotypic plasticity. The association between molecular markers and phenotypes is a general tendency in studies of the divergence of neutral traits and traits under selection [20,21,73]. It can be caused by different evolutionary mechanisms. First, it can be caused by a genetic linkage between molecular markers and loci under selection, resulting in genetic hitch-hiking of the molecular markers [85,86]. In our study, this is unlikely, given that microsatellite markers are putatively neutral, and have in general low genome coverage. Isolation by distance (IBD) and isolation by adaptation (IBA) can also result in simultaneous population differentiation in molecular markers and phenotypic traits. In the case of IBD, the differentiation cause is genetic drift, which acts on neutral markers as well as on loci under selection [20]. In the case of isolation by adaptation, the selective elimination of locally maladapted genotypes in the population causes a barrier to gene flow that will differentiate the whole genome, although the differentiation should be less pronounced in molecular markers than in traits under selection [73]. Under IBA, population genetic differentiation at molecular markers should be positively correlated with genetic differentiation at loci under selection, and both should be positively correlated to selection intensity. The COA and Mantel test results are consistent with this observation, suggesting that IBA might be responsible for the observed patterns of covariation between molecular markers and phenotypic traits. This conclusion should be considered with caution given that our results show only weak divergent selection among populations. Thus the observed patterns can also be partly due to IBD. However, no evidence of IBD was found for these populations based on Mantel tests for correlation between genetic and geographic distances. Furthermore, even low levels of population differentiation can contribute to variation in allelic frequencies. In coniferous tree species, for instance, neutral *F*_*ST*_ below 5% can still generate strong biases in genetic association or environmental association analyses neglecting this structure [87].

Regardless of the causes of the observed associations, the information it provides can be used to identify individuals in the populations of this study bearing traits with specific values without going through the lengthy process of phenotyping. Since microsatellites are neutral molecular markers, a statistical association between adaptive traits and microsatellite markers is not informative about the genetic basis of the adaptive trait. However, establishing a relationship between molecular markers, phenotypic traits and environmental variation would help to easily identify genotypes and phenotypes in the studied populations that can be of interest for the future exploration of the response of the studied populations to climate change, by helping to select for appropriate genotypes (e.g. genotypes with high plasticity, or with specific phenotypic values) to test their response to climate variation. Although the observed marker-trait associations are only relevant for the populations of this study, the same methods can be used to identify analogous associations in other populations, and thus select for new genotypes that have comparable trait associations as those in our study.

### Adaptive response of *F*. *rubra* to current and future climate change

The alpine populations of this study were overall highly differentiated from boreal and subalpine populations, and among the alpine populations, the population from the wettest habitat was the most genetically and phenotypically distinct. These results are somewhat consistent with an adaptive response to climate, although other environmental factors should not be overlooked. Indeed, we showed that locality explains between 30 and 50% more of the shared inertia than temperature and precipitation combined, so other factors, unaccounted for in this study also contribute and can be more important for shaping the observed patterns.

Finally, according to the climatic predictions for western Norway in the next century, the present populations of *F*. *rubra* will face an increasingly hotter and wetter environment [88], which based on the results of other studies on the same system is less selective than colder and drier environments [27]. Alpine populations could, therefore, benefit from the new, more favourable climatic conditions and increase their local abundance. Provided that warmer and wetter climate is favourable even beyond the climate values tested in this study, boreal populations could also benefit from climate change. Although it is also possible that *F*. *rubra* has an upper limit tolerance of temperature and moisture given that the species grows in considerably more temperate environments than Norway, it is unlikely that the boreal populations will face climate restrictions in the foreseeable future. Nevertheless, to reliably predict the response of boreal populations, we need data of their performance in temperature and precipitation conditions that are warmer and wetter than those tested in our study.

In conclusion, quantitative genetic differentiation between populations of *F*. *rubra* is consistent with the effect of divergent selection that is at least partly due to climate. The variation in adaptive traits and their plasticity is also correlated with variation in neutral molecular markers, and the covariation patterns are themselves influenced by variation in climate. Regardless of the causes of the statistical association between quantitative traits and molecular markers, the information it provides can be used to identify individuals bearing traits with specific values within our studied system without going through the lengthy process of phenotyping.

## Supporting information

S1 Fig Map of the populations used in this study. (PDF)

S2 Fig Results of coinertia analyses between trait values and molecular markers (A, B, C) and phenotypic plasticity and molecular markers (D, E, F). A. and D. Population projection on the first two COA axes. The grey arrows represent the PCA projections of trait values (beginning of arrow) and molecular markers (end of arrow). B. and E. COA axes loadings for phenotypic variables. Black lines–growth related trait means, bold lines–resource acquisition trait means, dashed lines–physiological trait means.φP0– maximum quantum yield of primary PS II photochemistry, PIABS–performance index for energy conservation from photons absorbed by PS II antenna. C. and F. COA axes loadings for genotypic structure. For clarity of the representation, only 15 alleles that have the highest loadings for either axis are shown. Full black lines–locus HVM3, dotted black lines– B3-B8, full grey lines– B4-D9, dotted grey lines–HVM2.
(PDF)

S3 Fig Experimental design. (PDF)

S1 Table Characteristics of the sampled area for the natural populations of the study. The dimensions are calculated as the distance between the most distant collecting points at two perpendicular directions.
(PDF)

S2 Table A. MANOVA results of the multivariate analysis of variance of the effect of selected alleles on trait values and plasticities. B. Regression coefficients of individual ANOVAs testing the effect of allelic presence/absence on trait mean and on plasticity.
(PDF)

S3 Table Estimates of within and between population variance for trait values and plasticities between population phenotypic variance (population variance),within population phenotypic variance (residual variance), between population additive genetic variance estimated from the animal model, within population additive genetic variance estimated from the animal model. (PDF)

S4 Table Genetic and genotypic diversity of the studied *Festuca rubra* populations. (PDF)

S5 Table Original genetic and phenotypic data used in this study. (XLSX)

## References

[1] Drake JM. Population effects of increased climate variation. Proc Biol Sci. 2005;272: 1823–1827. doi: 10.1098/rspb.2005.3148 16096095 10.1098/rspb.2005.3148 PMC1559868

[2] McCarty JP. Ecological Consequences of Recent Climate Change. Conserv Biol. 2001;15: 320–331. doi: 10.1046/j.1523-1739.2001.015002320.x

[3] Hoffmann AA, Sgrò CM. Climate change and evolutionary adaptation. Nature. 2011;470: 479–485. doi: 10.1038/nature09670 21350480 10.1038/nature09670

[4] Chen I-C, Hill JK, Ohlemüller R, Roy DB, Thomas CD. Rapid range shifts of species associated with high levels of climate warming. Science. 2011;333: 1024–1026. doi: 10.1126/science.1206432 21852500 10.1126/science.1206432

[5] Franks SJ, Weber JJ, Aitken SN. Evolutionary and plastic responses to climate change in terrestrial plant populations. Evol Appl. 2014;7: 123–139. doi: 10.1111/eva.12112 24454552 10.1111/eva.12112 PMC3894902

[6] Loarie SR, Duffy PB, Hamilton H, Asner GP, Field CB, Ackerly DD. The velocity of climate change. Nature. 2009;462: 1052–1055. doi: 10.1038/nature08649 20033047 10.1038/nature08649

[7] Kawecki TJ, Ebert D. Conceptual issues in local adaptation. Ecol Lett. 2004;7: 1225–1241.

[8] Pauls SU, Nowak C, Bálint M, Pfenninger M. The impact of global climate change on genetic diversity within populations and species. Mol Ecol. 2013;22: 925–946. doi: 10.1111/mec.12152 23279006 10.1111/mec.12152

[9] Sultan SE. Phenotypic plasticity and plant adaptation. Acta Bot Neerlandica. 1995;44: 363–383.

[10] Nicotra AB, Atkin OK, Bonser SP, Davidson AM, Finnegan EJ, Mathesius U, et al Plant phenotypic plasticity in a changing climate. Trends Plant Sci. 2010;15: 684–692. doi: 10.1016/j.tplants.2010.09.008 20970368 10.1016/j.tplants.2010.09.008

[11] Schlichting CD. The Evolution of Phenotypic Plasticity in Plants. Annu Rev Ecol Syst. 1986;17: 667–693. doi: 10.1146/annurev.es.17.110186.003315

[12] Via S. Adaptive phenotypic plasticity: target or by-product of selection in a variable environment? Am Nat. 1993;142: 352–365. doi: 10.1086/285542 19425981 10.1086/285542

[13] Springate DA, Scarcelli N, Rowntree J, Kover PX. Correlated response in plasticity to selection for early flowering in Arabidopsis thaliana. J Evol Biol. 2011;24: 2280–2288. doi: 10.1111/j.1420-9101.2011.02360.x 21812854 10.1111/j.1420-9101.2011.02360.x

[14] Davis MB, Shaw RG, Etterson JR. Evolutionary Responses to Changing Climate. Ecology. 2005;86: 1704–1714. doi: 10.1890/03-0788

[15] Pickett STA. Space-for-Time Substitution as an Alternative to Long-Term Studies Long-Term Studies in Ecology. Springer, New York, NY; 1989 pp. 110–135. doi: 10.1007/978-1-4615-7358-6_5

[16] Blois JL, Williams JW, Fitzpatrick MC, Jackson ST, Ferrier S. Space can substitute for time in predicting climate-change effects on biodiversity. Proc Natl Acad Sci. 2013;110: 9374–9379. doi: 10.1073/pnas.1220228110 23690569 10.1073/pnas.1220228110 PMC3677423

[17] Lester RE, Close PG, Barton JL, Pope AJ, Brown SC. Predicting the likely response of data-poor ecosystems to climate change using space-for-time substitution across domains. Glob Change Biol. 2014;20: 3471–3481. 10.1111/gcb.12634 24832685

[18] Meerhoff M. Environmental Warming in Shallow Lakes. A Review of Potential Changes in Community Structure as Evidenced from Space-for-Time Substitution Approaches. 2012;

[19] Merilä J, Hendry AP. Climate change, adaptation, and phenotypic plasticity: the problem and the evidence. Evol Appl. 2014;7: 1–14. doi: 10.1111/eva.12137 24454544 10.1111/eva.12137 PMC3894893

[20] Merilä J, Crnokrak P. Comparison of genetic differentiation at marker loci and quantitative traits. J Evol Biol. 2001;14: 892–903.

[21] Leinonen T, McCairns RS, O’Hara RB, Merilä J. QST–FST comparisons: evolutionary and ecological insights from genomic heterogeneity. Nat Rev Genet. 2013;14: 179–190. doi: 10.1038/nrg3395 23381120 10.1038/nrg3395

[22] Karhunen M, Ovaskainen O, Herczeg G, Merilä J. Bringing habitat information into statistical tests of local adaptation in quantitative traits: a case study of nine-spined sticklebacks. Evolution. 2014;68: 559–568. doi: 10.1111/evo.12268 24117061 10.1111/evo.12268

[23] Li S-L, Vasemägi A, Ramula S. Genetic variation facilitates seedling establishment but not population growth rate of a perennial invader. Ann Bot. 2015; mcv145. 10.1093/aob/mcv145 PMC4701146 26420202

[24] Luo Y, Widmer A, Karrenberg S. The roles of genetic drift and natural selection in quantitative trait divergence along an altitudinal gradient in Arabidopsis thaliana. Heredity. 2015;114: 220–228. doi: 10.1038/hdy.2014.89 25293874 10.1038/hdy.2014.89 PMC4815633

[25] Skálová H, Pecháčková S, Suzuki J, Herben T, Hara T, Hadincová V, et al Within population genetic differentiation in traits affecting clonal growth: Festuca rubra in a mountain grassland. J Evol Biol. 1997;10: 383–406. doi: 10.1046/j.1420-9101.1997.10030383.x

[26] Herben T, Krahulec F, Hadincová V, Pecháčková S. Clone-specific response of Festuca rubra to natural variation in biomass and species composition of neighbours. Oikos. 2001;95: 43–52.

[27] Münzbergová Z, Hadincová V, Skálová H, Vandvik V. Genetic differentiation and plasticity interact along temperature and precipitation gradients to determine plant performance under climate change. J Ecol. 2017; doi: 10.1111/1365-2745.12762

[28] Meineri E, Skarpaas O, Spindelböck J, Bargmann T, Vandvik V. Direct and size-dependent effects of climate on flowering performance in alpine and lowland herbaceous species. J Veg Sci. 2014;25: 275–286. doi: 10.1111/jvs.12062

[29] Ellstrand NC, Roose ML. Patterns of genotypic diversity in clonal plant species. Am J Bot. 1987;74: 123–131. doi: 10.2307/2444338

[30] Klanderud K, Vandvik V, Goldberg D. The importance of biotic vs. abiotic drivers of local plant community composition along regional bioclimatic gradients. PloS One. 2015;10: e0130205 doi: 10.1371/journal.pone.0130205 26091266 10.1371/journal.pone.0130205 PMC4474800

[31] Castro S, Münzbergová Z, Raabová J, Loureiro J. Breeding barriers at a diploid–hexaploid contact zone in Aster amellus. Evol Ecol. 2010;25: 795–814. doi: 10.1007/s10682-010-9439-5

[32] Govindjee SA. On the relation between the Kautsky effect (Chlorophyll a fluorescence induction) and photosystem II: Basic and applications of the OJIP fluorescence transient. J Photoch Photobio B. 2011;104: 236–257. 10.1016/j.jphotobiol.2010.12.010 21295993

[33] Valladares F, Martinez-Ferri E, Balaguer L, Perez-Corona E, Manrique E. Low leaf-level response to light and nutrients in Mediterranean evergreen oaks: a conservative resource-use strategy? New Phytol. 2000;148: 79–91. 10.1046/j.1469-8137.2000.00737.x 33863045

[34] Valladares F, Sanchez-Gomez D, Zavala MA. Quantitative estimation of phenotypic plasticity: bridging the gap between the evolutionary concept and its ecological applications. J Ecol. 2006;94: 1103–1116. doi: 10.1111/j.1365-2745.2006.01176.x

[35] FU Y-B, Qiu J, Peterson GW, Willms WD, Wilmshurst JF. Characterization of microsatellite markers for rough fescue species (Festuca spp.). Mol Ecol Resour. 2006;6: 894–896.

[36] Lauvergeat V, Barre P, Bonnet M, Ghesquiere M. Sixty simple sequence repeat markers for use in the Festuca–Lolium complex of grasses. Mol Ecol Resour. 2005;5: 401–405.

[37] R Core Team. R: A language and environment for statistical computing. R Foundation for Statistical Computing, Vienna, Austria 2014;2014 Available: http://www.R-project.org/.

[38] Porth I, Klapste J, McKown AD, La Mantia J, Guy RD, Ingvarsson PK, et al Evolutionary Quantitative Genomics of Populus trichocarpa. Plos One. 2015;10: e0142864 doi: 10.1371/journal.pone.0142864 26599762 10.1371/journal.pone.0142864 PMC4658102

[39] Henderson CR. Applications of Linear Models in Animal Breeding. University of Guelph; 1984.

[40] Butler DG, Cullis BR, Gilmour AR, Gogel BJ. ASReml-R reference manual. State Qld Dep Prim Ind Fish Brisb. 2009; Available: http://discoveryfoundation.org.uk/downloads/asreml/release3/asreml-R.pdf

[41] Loiselle BA, Sork VL, Nason J, Graham C. Spatial genetic structure of a tropical understory shrub, Psychotria officinalis (Rubiaceae). Am J Bot. 1995; 1420–1425.

[42] Hardy OJ, Vekemans X. SPAGeDi: a versatile computer program to analyse spatial genetic structure at the individual or population levels. Mol Ecol Resour. 2002;2: 618–620.

[43] Leinonen T, Cano JM, Mäkinen H, Merilä J. Contrasting patterns of body shape and neutral genetic divergence in marine and lake populations of threespine sticklebacks. J Evol Biol. 2006;19: 1803–1812. doi: 10.1111/j.1420-9101.2006.01182.x 17040377 10.1111/j.1420-9101.2006.01182.x

[44] Brommer JE. Whither PST? The approximation of QST by PST in evolutionary and conservation biology. J Evol Biol. 2011;24: 1160–1168. doi: 10.1111/j.1420-9101.2011.02268.x 21457173 10.1111/j.1420-9101.2011.02268.x

[45] Whitlock MC, Guillaume F. Testing for Spatially Divergent Selection: Comparing QST to FST. Genetics. 2009;183: 1055–1063. doi: 10.1534/genetics.108.099812 19687138 10.1534/genetics.108.099812 PMC2778959

[46] Lind MI, Ingvarsson PK, Johansson H, Hall D, Johansson F. Gene Flow and Selection on Phenotypic Plasticity in an Island System of Rana Temporaria. Evolution. 2011;65: 684–697. doi: 10.1111/j.1558-5646.2010.01122.x 20825480 10.1111/j.1558-5646.2010.01122.x

[47] Lewontin RC, Krakauer J. Distribution of Gene Frequency as a Test of the Theory of the Selective Neutrality of Polymorphisms. Genetics. 1973;74: 175–195. 4711903 10.1093/genetics/74.1.175 PMC1212935

[48] Michalakis Y, Excoffier L. A generic estimation of population subdivision using distances between alleles with special reference for microsatellite loci. Genetics. 1996;142: 1061–1064. 8849912 10.1093/genetics/142.3.1061 PMC1207006

[49] Edelaar PIM, Burraco P, GOMEZ-MESTRE I. Comparisons between QST and FST—how wrong have we been? Mol Ecol. 2011;20: 4830–4839. doi: 10.1111/j.1365-294X.2011.05333.x 22060729 10.1111/j.1365-294X.2011.05333.x

[50] Ellegren H. Microsatellites: simple sequences with complex evolution. Nat Rev Genet. 2004;5: 435–445. doi: 10.1038/nrg1348 15153996 10.1038/nrg1348

[51] Dolédec S, Chessel D. Co-inertia analysis: an alternative method for studying species–environment relationships. Freshw Biol. 1994;31: 277–294.

[52] Dray S, Chessel D, Thioulouse J. Co-inertia analysis and the linking of ecological data tables. Ecology. 2003;84: 3078–3089.

[53] Jarraud S, Mougel C, Thioulouse J, Lina G, Meugnier H, Forey F, et al Relationships between Staphylococcus aureus Genetic Background, Virulence Factors, agr Groups (Alleles), and Human Disease. Infect Immun. 2002;70: 631–641. doi: 10.1128/IAI.70.2.631-641.2002 11796592 10.1128/IAI.70.2.631-641.2002 PMC127674

[54] Dray S, Dufour A-B, others. The ade4 package: implementing the duality diagram for ecologists. J Stat Softw. 2007;22: 1–20.

[55] Jombart T. adegenet: a R package for the multivariate analysis of genetic markers. Bioinformatics. 2008;24: 1403–1405. doi: 10.1093/bioinformatics/btn129 18397895 10.1093/bioinformatics/btn129

[56] Clark LV, Jasieniuk M. POLYSAT: an R package for polyploid microsatellite analysis. Mol Ecol Resour. 2011;11: 562–566. doi: 10.1111/j.1755-0998.2011.02985.x 21481215 10.1111/j.1755-0998.2011.02985.x

[57] Esselink GD, Nybom H, Vosman B. Assignment of allelic configuration in polyploids using the MAC-PR (microsatellite DNA allele counting-peak ratios) method. TAG Theor Appl Genet Theor Angew Genet. 2004;109: 402–408. doi: 10.1007/s00122-004-1645-5 15085263 10.1007/s00122-004-1645-5

[58] Dufresne F, Stift M, Vergilino R, Mable BK. Recent progress and challenges in population genetics of polyploid organisms: an overview of current state-of-the-art molecular and statistical tools. Mol Ecol. 2014;23: 40–69. doi: 10.1111/mec.12581 24188632 10.1111/mec.12581

[59] Pons O, Petit RJ. Measwring and testing genetic differentiation with ordered versus unordered alleles. Genetics. 1996;144: 1237–1245. 8913764 10.1093/genetics/144.3.1237 PMC1207615

[60] Motro U, Thomson G. On heterozygosity and the effective size of populations subject to size changes. Evolution. 1982;36: 1059–1066. doi: 10.1111/j.1558-5646.1982.tb05474.x 28567820 10.1111/j.1558-5646.1982.tb05474.x

[61] Ohta T, Kimura M. A model of mutation appropriate to estimate the number of electrophoretically detectable alleles in a finite population. Genet Res. 1973;22: 201–204. 4777279 10.1017/s0016672300012994

[62] Hetherington AM, Woodward FI. The role of stomata in sensing and driving environmental change. Nature. 2003;424: 901 doi: 10.1038/nature01843 12931178 10.1038/nature01843

[63] Zhang L, Niu H, Wang S, Zhu X, Luo C, Li Y, et al Gene or environment? Species-specific control of stomatal density and length. Ecol Evol. 2012;2: 1065–1070. doi: 10.1002/ece3.233 22837850 10.1002/ece3.233 PMC3399171

[64] Raven JA. Selection pressures on stomatal evolution. New Phytol. 2002;153: 371–386. doi: 10.1046/j.0028-646X.2001.00334.x 10.1046/j.0028-646X.2001.00334.x 33863217

[65] Beerling DJ, Chaloner WG, Huntley B, Pearson JA, Tooley MJ. Stomatal density responds to the glacial cycle of environmental change. Proc R Soc Lond B. 1993;251: 133–138. doi: 10.1098/rspb.1993.0019

[66] Aykanat T, Johnston SE, Orell P, Niemelä E, Erkinaro J, Primmer CR. Low but significant genetic differentiation underlies biologically meaningful phenotypic divergence in a large Atlantic salmon population. Mol Ecol. 2015;24: 5158–5174. doi: 10.1111/mec.13383 26363183 10.1111/mec.13383

[67] García-Navas V, Ferrer ES, Sanz JJ, Ortego J. The role of immigration and local adaptation on fine-scale genotypic and phenotypic population divergence in a less mobile passerine. J Evol Biol. 2014;27: 1590–1603. doi: 10.1111/jeb.12412 24890737 10.1111/jeb.12412

[68] Gömöry D, Ditmarová L, Hrivnák M, Jamnická G, Kmet’ J, Krajmerová D, et al Differentiation in phenological and physiological traits in European beech (Fagus sylvatica L.). Eur J For Res. 2015;134: 1075–1085.

[69] Michalski SG, Durka W. Separation in flowering time contributes to the maintenance of sympatric cryptic plant lineages. Ecol Evol. 2015;5: 2172–2184. doi: 10.1002/ece3.1481 26078854 10.1002/ece3.1481 PMC4461419

[70] Pujol B, Wilson AJ, Ross RIC, Pannell JR. Are QST–FST comparisons for natural populations meaningful? Mol Ecol. 2008;17: 4782–4785. doi: 10.1111/j.1365-294X.2008.03958.x 19140971 10.1111/j.1365-294X.2008.03958.x

[71] Münzbergová Z, Hadincová V. Transgenerational plasticity as an important mechanism affecting response of clonal species to changing climate. Ecol Evol. 2017; Available: http://onlinelibrary.wiley.com/doi/10.1002/ece3.3105/full 10.1002/ece3.3105 PMC5528211 28770062

[72] McKay JK, Latta RG. Adaptive population divergence: markers, QTL and traits. Trends Ecol Evol. 2002;17: 285–291. doi: 10.1016/S0169-5347(02)02478-3

[73] Nosil P, Funk DJ, Ortiz-Barrientos D. Divergent selection and heterogeneous genomic divergence. Mol Ecol. 2009;18: 375–402. doi: 10.1111/j.1365-294X.2008.03946.x 19143936 10.1111/j.1365-294X.2008.03946.x

[74] Gonzalo-Turpin H, Hazard L. Local adaptation occurs along altitudinal gradient despite the existence of gene flow in the alpine plant species Festuca eskia. J Ecol. 2009;97: 742–751. doi: 10.1111/j.1365-2745.2009.01509.x

[75] Körner C, Neumayer M, Menendez-Riedl SP, Smeets-Scheel A. Functional morphology of mountain plants. Flora. 1989;182: 353–383.

[76] Stenström A, Jónsdóttir IS, Augner M. Genetic and environmental effects on morphology in clonal sedges in the Eurasian Arctic. Am J Bot. 2002;89: 1410–1421. doi: 10.3732/ajb.89.9.1410 21665742 10.3732/ajb.89.9.1410

[77] Debat V, David P. Mapping phenotypes: canalization, plasticity and developmental stability. Trends Ecol Evol. 2001;16: 555–561.

[78] Nicotra AB, Segal DL, Hoyle GL, Schrey AW, Verhoeven KJF, Richards CL. Adaptive plasticity and epigenetic variation in response to warming in an Alpine plant. Ecol Evol. 2015;5: 634–647. doi: 10.1002/ece3.1329 25691987 10.1002/ece3.1329 PMC4328768

[79] De Witte LC, Stöcklin J. Longevity of clonal plants: why it matters and how to measure it. Ann Bot. 2010;106: 859–870. doi: 10.1093/aob/mcq191 20880935 10.1093/aob/mcq191 PMC2990663

[80] Ahmad P, Prasad MNV. Abiotic stress responses in plants: metabolism, productivity and sustainability. Springer Science & Business Media; 2011.

[81] Brommer JE, Merilä J, Sheldon BC, Gustafsson L. Natural selection and genetic variation for reproductive reaction norms in a wild bird population. Evol Int J Org Evol. 2005;59: 1362–1371. 16050111

[82] Rogell B, Dannewitz J, Palm S, Petersson E, Dahl J, Prestegaard T, et al Strong divergence in trait means but not in plasticity across hatchery and wild populations of sea-run brown trout Salmo trutta. Mol Ecol. 2012;21: 2963–2976. doi: 10.1111/j.1365-294X.2012.05590.x 22548410 10.1111/j.1365-294X.2012.05590.x

[83] De Kort H, Vander Mijnsbrugge K, Vandepitte K, Mergeay J, Ovaskainen O, Honnay O. Evolution, plasticity and evolving plasticity of phenology in the tree species Alnus glutinosa. J Evol Biol. 2016;29: 253–264. doi: 10.1111/jeb.12777 26484499 10.1111/jeb.12777

[84] De Jong G. Evolution of phenotypic plasticity: patterns of plasticity and the emergence of ecotypes. New Phytol. 2005;166: 101–118. doi: 10.1111/j.1469-8137.2005.01322.x 15760355 10.1111/j.1469-8137.2005.01322.x

[85] Kim Y, Stephan W. Joint Effects of Genetic Hitchhiking and Background Selection on Neutral Variation. Genetics. 2000;155: 1415–1427. 10880499 10.1093/genetics/155.3.1415 PMC1461159

[86] Barton NH. Genetic hitchhiking. Philos Trans R Soc Lond B Biol Sci. 2000;355: 1553–1562. doi: 10.1098/rstb.2000.0716 11127900 10.1098/rstb.2000.0716 PMC1692896

[87] Mosca E, Eckert AJ, Di Pierro EA, Rocchini D, La Porta N, Belletti P, et al The geographical and environmental determinants of genetic diversity for four alpine conifers of the European Alps. Mol Ecol. 2012;21: 5530–5545. doi: 10.1111/mec.12043 23058000 10.1111/mec.12043

[88] Hanssen-Bauer I, Achberger C, Benestad RE, Chen D, Førland EJ. Statistical downscaling of climate scenarios over Scandinavia. Clim Res. 2005;29: 255–268.

