# Supplementary material for: Adaptive differentiation of Festuca rubra along a climate gradient revealed by molecular markers and quantitative traits
Source: PLoS One. 2018 Apr 4;13(4):e0194670. doi: 10.1371/journal.pone.0194670 (PMC5884518; doi:10.1371/journal.pone.0194670)

Supporting Information

Adaptive differentiation of *Festuca rubra* along a climate gradient revealed by molecular markers and quantitative traits

PLOS One

Bojana Stojanova[*,1,2], Mária Šurinová[1,2], Jaroslav Klápště[3], Veronika Kolaríková[1], Věroslava Hadincová[2], Zuzana Münzbergová [1,2]
[1] Department of Botany, Faculty of Science, Charles University, Prague, Czech Republic
[2] Institute of Botany, Academy of Sciences of the Czech Republic, Průhonice, Czech Republic
[3] Scion (New Zealand Forest Research Institute Ltd.), Whakarewarewa, Rotorua, 3046, New Zealand
[*] Corresponding author: bojana.stojanova@gmail.com, tel. +420 271 015 708, Fax +420 271 015 105

S1 Figure. Map of the populations used in the study

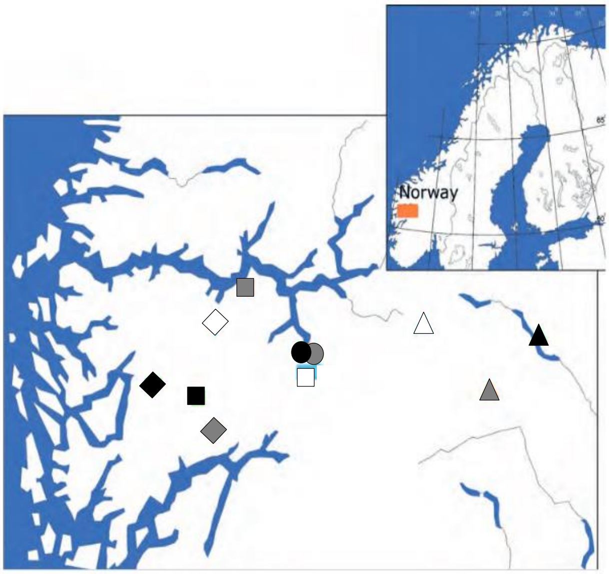

Populations environment of origin

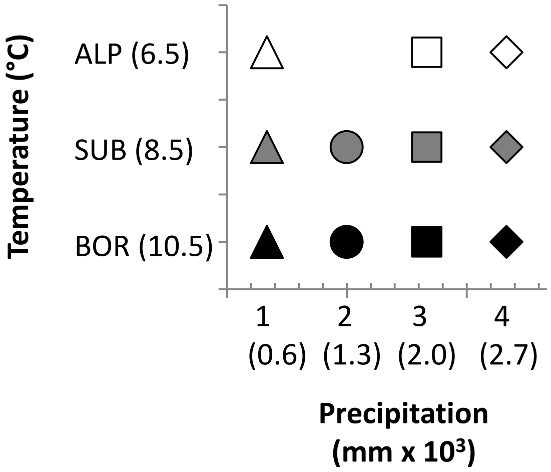

Supplement: S1 Fig — (PDF) [file pone.0194670.s001.pdf]
