# Supplementary material for: Adaptive differentiation of Festuca rubra along a climate gradient revealed by molecular markers and quantitative traits
Source: PLoS One. 2018 Apr 4;13(4):e0194670. doi: 10.1371/journal.pone.0194670 (PMC5884518; doi:10.1371/journal.pone.0194670)
Supplement: S2 Fig — A. and D. Population projection on the first two COA axes. The grey arrows represent the PCA projections of trait values (beginning of arrow) and molecular markers (end of arrow). B. and E. COA axes loadings for phenotypic variables. Black lines–growth related trait means, bold lines–resource acquisition trait means, dashed lines–physiological trait means.φP0– maximum quantum yield of primary PS II photochemistry, PIABS–performance index for energy conservation from photons absorbed by PS II antenna. C. and F. COA axes loadings for genotypic structure. For clarity of the representation, only 15 alleles that have the highest loadings for either axis are shown. Full black lines–locus HVM3, dotted black lines– B3-B8, full grey lines– B4-D9, dotted grey lines–HVM2. (PDF) [file pone.0194670.s002.pdf]

SUPPORTING INFORMATION

Adaptive differentiation of *Festuca rubra* along a climate gradient revealed by molecular markers and quantitative traits

PLOS One

Bojana Stojanova[*,1,2],Mária Šurinová[1,2], Jaroslav Klápště[3], Veronika Koláriková[1], Věroslava Hadincová[2], Zuzana Münzbergová [1,2]

[1] Department of Botany, Faculty of Science, Charles University, Prague, Czech Republic

[2] Institute of Botany, Academy of Sciences of the Czech Republic, Průhonice, Czech Republic

[3] Scion (New Zealand Forest Research Institute Ltd.), Whakarewarewa, Rotorua, 3046, New Zealand

[*] Corresponding author: bojana.stojanova@gmail.com, tel. +420 271 015 708, Fax +420 271 015 105

S2 Figure. Results of coinertia analyses between trait values and molecular markers (A, B, C) and phenotypic plasticity and molecular markers (D, E, F).

A. and D. Population projection on the first two COA axes. The grey arrows represent the PCA projections of trait values (beginning of arrow) and molecular markers (end of arrow).

B. and E. COA axes loadings for phenotypic variables. Black lines – growth related trait means, bold lines – resource acquisition trait means, dashed lines – physiological trait means.ϕP0– maximum quantum yield of primary PS II photochemistry, PIABS –performance index for energy conservation from photons absorbed by PS II antenna.

C. and F. COA axes loadings for genotypic structure. For clarity of the representation, only 15 alleles that have the highest loadings for either axis are shown. Full black lines – locus HVM3, dotted black lines – B3-B8, full grey lines – B4-D9, dotted grey lines – HVM2.

S2 Figure

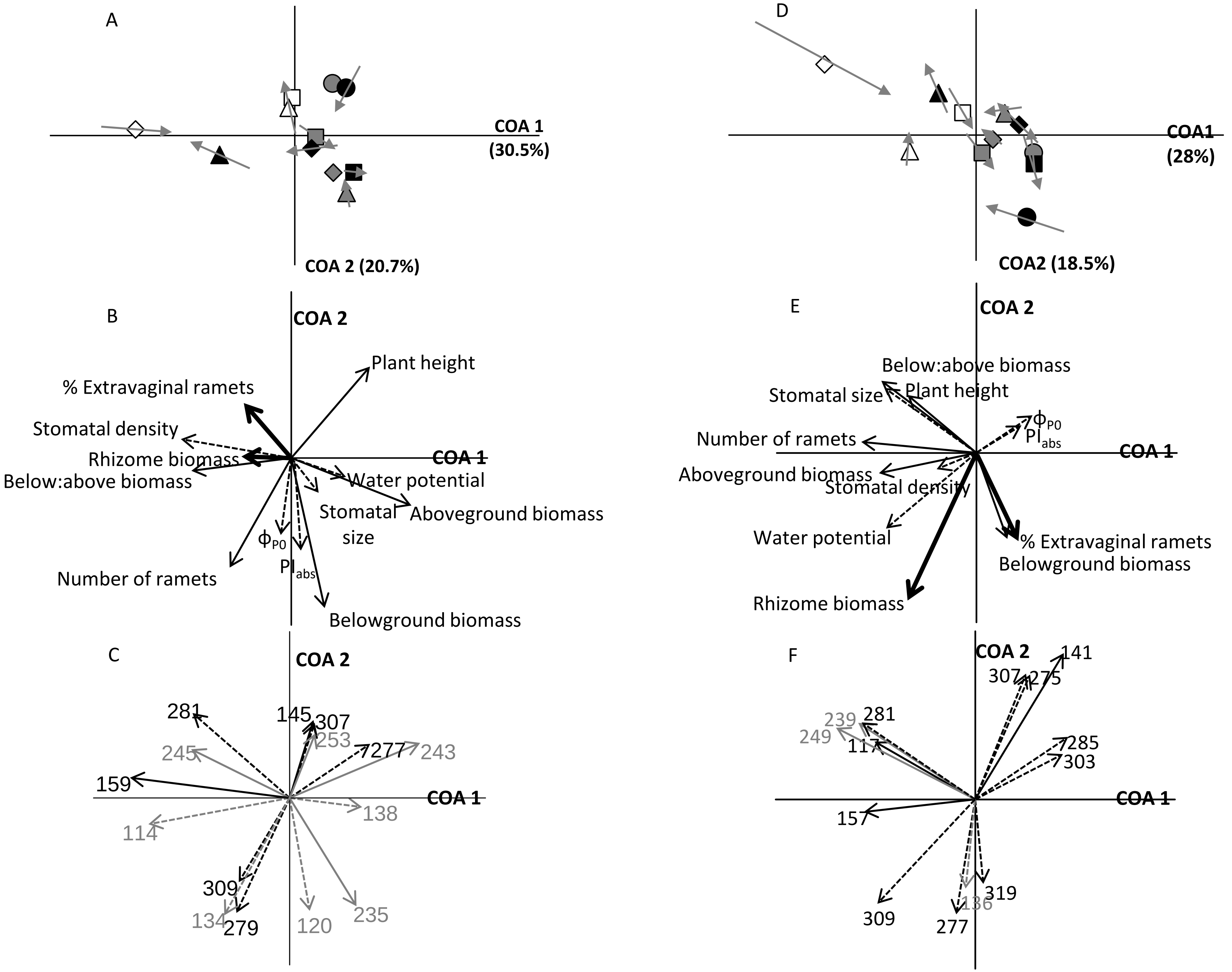

A
COA 1 (30.5%)
COA 2 (20.7%)
B
COA 2
COA 1
Plant height
% Extravaginal ramets
Stomatal density
Rhizome biomass
Below:above biomass
Water potential
Stomatal size
Aboveground biomass
ϕ_{P0}
PI_{abs}
Number of ramets
Belowground biomass
C
COA 2
COA 1
281
145
307
253
277
243
245
159
138
114
309
134
279
120
235
D
COA1 (28%)
COA2 (18.5%)
E
COA 2
COA 1
Below:above biomass
Stomatal size
Plant height
ϕ_{P0}
PI_{abs}
Number of ramets
Aboveground biomass
Stomatal density
Water potential
% Extravaginal ramets
Belowground biomass
Rhizome biomass
F
COA 2
COA 1
141
307
275
239
281
249
117
285
303
157
309
277
136
319
