# Supplementary material for: Adaptive differentiation of Festuca rubra along a climate gradient revealed by molecular markers and quantitative traits
Source: PLoS One. 2018 Apr 4;13(4):e0194670. doi: 10.1371/journal.pone.0194670 (PMC5884518; doi:10.1371/journal.pone.0194670)
Supplement: S1 Table — The dimensions are calculated as the distance between the most distant collecting points at two perpendicular directions. (PDF) [file pone.0194670.s004.pdf]

SUPPORTING INFORMATION

Adaptive differentiation of *Festuca rubra* along a climate gradient revealed by molecular markers and quantitative traits

PLOS One

Bojana Stojanova[*,1,2], Mária Šurinová[1,2], Jaroslav Klápště[3], Veronika Kolaříková[1], Věroslava Hadincová[2], Zuzana Münzbergová [1,2]

[1] Department of Botany, Faculty of Science, Charles University, Prague, Czech Republic

[2] Institute of Botany, Academy of Sciences of the Czech Republic, Průhonice, Czech Republic

[3] Scion (New Zealand Forest Research Institute Ltd.), Whakarewarewa, Rotorua, 3046, New Zealand

[*] Corresponding author: bojana.stojanova@gmail.com, tel. +420 271 015 708, Fax +420 271 015 105

S1 Table. Characteristics of the sampled area for the natural populations of the study. The dimensions are calculated as the distance between the most distant collecting points at two perpendicular directions

Population	plot length a (m)	plot lenghth b (m)	collecting area (m2)
ALP1	40	35	1400
ALP2	55	45	2475
ALP3	22	22	484
ALP4	10	10	100
BOR1	10	10	100
BOR2	47	1	47
BOR3	93	92	8556
BOR4	26	20	520
SUB1	29	29	841
SUB2	78	32	2496
SUB3	45	40	1800
SUB4	55	48	2640
