# Supplementary material for: Adaptive differentiation of Festuca rubra along a climate gradient revealed by molecular markers and quantitative traits
Source: PLoS One. 2018 Apr 4;13(4):e0194670. doi: 10.1371/journal.pone.0194670 (PMC5884518; doi:10.1371/journal.pone.0194670)
Supplement: S2 Table — A. MANOVA results of the multivariate analysis of variance of the effect of selected alleles on trait values and plasticities. B. Regression coefficients of individual ANOVAs testing the effect of allelic presence/absence on trait mean and on plasticity. (PDF) [file pone.0194670.s005.pdf]

**Supporting Information**
Adaptive differentiation of *Festuca rubra* along a climate gradient revealed by molecular markers and quantitative traits
*PLOS One*
Bojana Stojanova[*,1,2],Mária Šurinová[1,2], Jaroslav Klápště[3], Veronika Koláriková[1], Věroslava Hadincová[2], Zuzana Münzbergová [1,2]
[1] Department of Botany, Faculty of Science, Charles University, Prague, Czech Republic
[2] Institute of Botany, Academy of Sciences of the Czech Republic, Průhonice, Czech Republic
[3] Scion (New Zealand Forest Research Institute Ltd.), Whakarewarewa, Rotorua, 3046, New Zealand
[*] Corresponding author: bojana.stojanova@gmail.com, tel. +420 271 015 708, Fax +420 271 015 105

**S2 Table. A.** MANOVA results of the multivariate analysis of variance of the effect of selected alleles on trait values and plasticities. **B.** Regression coefficients of individual ANOVAs testing the effect of allelic presence/absence on trait mean and on plasticity.

A.

| | Means MANOVA | | Plasticity MANOVA | |
|---|---|---|---|---|
| Allele | Pillai | p-value | Pillai | p-value |
| HVM2.134 | **0.378** | **0.004** | NT | NT |
| HVM2.130 | 0.205 | 0.291 | 0.157 | 0.173 |
| HVM2.124 | 0.134 | 0.710 | NT | NT |
| HVM3.159 | **0.415** | **0.001** | 0.067 | 0.877 |
| HVM3.151 | **0.334** | **0.014** | 0.136 | 0.302 |
| B3_B8.321 | **0.305** | **0.033** | NT | NT |
| B3_B8.283 | 0.169 | 0.489 | NT | NT |
| B3_B8.281 | 0.187 | 0.383 | NT | NT |
| B3_B8.273 | **0.291** | **0.046** | NT | NT |
| B3_B8.277 | NT | NT | 0.119 | 0.431 |
| B3_B8.279 | **0.338** | **0.013** | 0.164 | 0.142 |
| B4_D9.243 | **0.354** | **0.008** | **0.188** | **0.067** |
| B4_D9.235 | **0.412** | **0.001** | NT | NT |

Significant values are in bold. NT – alleles that were not selected for the given model.

**Supporting Information**

Adaptive differentiation of *Festuca rubra* along a climate gradient revealed by molecular markers and quantitative traits

*PLOS One*

Bojana Stojanova[*,1,2], Mária Šurinová[1,2], Jaroslav Klápště[3], Veronika Koláriková[1], Věroslava Hadincová[2], Zuzana Münzbergová [1,2]

[1] Department of Botany, Faculty of Science, Charles University, Prague, Czech Republic

[2] Institute of Botany, Academy of Sciences of the Czech Republic, Průhonice, Czech Republic

[3] Scion (New Zealand Forest Research Institute Ltd.), Whakarewarewa, Rotorua, 3046, New Zealand

[*] Corresponding author: bojana.stojanova@gmail.com, tel. +420 271 015 708, Fax +420 271 015 105

B.

| | Locus | Leaf length | Number of ramets | % extravaginal ramets | Rhizome weight | Belowground weight | Aboveground weight | root/ shoot | ϕP0 | PIABS | Stomatal density | Stomatal size | Water potential |
|---|---|---|---|---|---|---|---|---|---|---|---|---|---|
| Trait mean | HVM2.134 | -0.52 | 0.89 | | | | | | 0.529 | | | | |
| | HVM2.130 | | | | | 0.485 | | | | | | | |
| | HVM2.124 | | | | | | -0.34 | | | | | | |
| | HVM3.159 | | | | 0.336 | | -0.85 | 0.241 | | 0.363 | 1.158 | | -0.45 |
| | HVM3.151 | -0.79 | | | | | | | | | | | |
| | B3_B8.321 | | | | 0.417 | | | | | | | | |
| | B3_B8.283 | | | 0.955 | 1.231 | | -0.78 | 0.909 | | | | | |
| | B3_B8.281 | | | | | | | 0.841 | | -0.39 | | | |
| | B3_B8.273 | | | | | | | | | -0.68 | 0.458 | | |
| | B3_B8.279 | -0.55 | 0.658 | | 0.763 | | | | 0.466 | | | | -0.47 |
| | B4_D9.243 | 0.753 | | -0.67 | -0.75 | | | -0.5 | -0.63 | -0.39 | | | |
| | B4_D9.235 | | | | -0.48 | 0.834 | 0.786 | | 0.945 | 0.98 | | | |
| Trait plasticity | HVM2.130 | | | | | | | | | | | | |
| | HVM3.159 | | | | | | | | | | | | |
| | HVM3.151 | | | | | | | | 0.441 | 0.571 | | | |
| | B3_B8.277 | | | | 0.531 | | | | | | | | 0.368 |
| | B3_B8.279 | | | 0.533 | | | | | -0.39 | | | | |
| | B4_D9.243 | | | | | 0.372 | | -0.49 | 0.445 | | -0.41 | | -0.35 |

Only coefficients that were statistically significant in either model are shown. ϕP0 – maximum quantum yield of primary PS II photochemistry, PIABS –performance index for energy conservation from photons absorbed by PS II antenna.
