# Supplementary material for: Adaptive differentiation of Festuca rubra along a climate gradient revealed by molecular markers and quantitative traits
Source: PLoS One. 2018 Apr 4;13(4):e0194670. doi: 10.1371/journal.pone.0194670 (PMC5884518; doi:10.1371/journal.pone.0194670)
Supplement: S3 Table — (PDF) [file pone.0194670.s006.pdf]

SUPPORTING INFORMATION

Adaptive differentiation of *Festuca rubra* along a climate gradient revealed by molecular markers and quantitative traits

PLOS One

Bojana Stojanova[*,1,2],Mária Šurinová[1,2], Jaroslav Klápště[3], Veronika Koláriková[1], Věroslava Hadincová[2], Zuzana Münzbergová [1,2]

[1] Department of Botany, Faculty of Science, Charles University, Prague, Czech Republic

[2] Institute of Botany, Academy of Sciences of the Czech Republic, Průhonice, Czech Republic

[3] Scion (New Zealand Forest Research Institute Ltd.), Whakarewarewa, Rotorua, 3046, New Zealand

[*] Corresponding author: bojana.stojanova@gmail.com, tel. +420 271 015 708, Fax +420 271 015 105

S3 Table. Estimates of within and between population variance for trait values and plasticites

	Trait				Plasticity			
	P_{ST}		Q_{ST}		P_{ST}		Q_{ST}	
	σ_b^2	σ_w^2	σ_p^2	σ_a^2	σ_b^2	σ_w^2	σ_p^2	σ_a^2
Height	1.381	14.733	1.249	5.861	0.0007	0.0124	0.0006	0.0002
Number of ramets	4.934	73.272	4.529	36.27	0.0004	0.0234	NA	NA
% extravaginal ramets	0.003	0.05	0.003	0.018	0.0006	0.0410	0.0001	0.004
Aboveground biomass	0.004	0.061	0.004	0.013	0.0024	0.0323	0.002	0.0007
Belowground biomass	0.019	0.296	NA	NA	0.0011	0.0235	NA	NA
Rhizome biomass	0.0001	0.003	0.0001	0.0009	<0.0001	0.0201	0	0.002
Below:aboveground biomass	0	18308.91	0.0044	0.003	0.0009	0.0244	0.0009	0
Fi.P0	0	0.001	0	0.0002	<0.0001	0.0048	0.0001	0.001
Pabs	0.004	0.075	0.004	0.022	0.0004	0.0143	0.0003	0.0003
Water potential	0.003	0.373	NA	NA	0.0263	0.5316	0.026	0.0001
Stomatal density	70.185	283.347	74.61	105.47	0.0016	0.0148	NA	NA
Stomatal size	0.8099	13.984	0.435	4.15	0.0002	0.0069	0.0003	0.0003

σ_b^2 – between population phenotypic variance (population variation)

σ_w^2- within population phenotypic variance (residual variation)

σ_p^2- between population additive genetic variance estimated from the animal model

σ_a^2 – within population additive genetic variance estimated from the animal model
