# Supplementary material for: Adaptive differentiation of Festuca rubra along a climate gradient revealed by molecular markers and quantitative traits
Source: PLoS One. 2018 Apr 4;13(4):e0194670. doi: 10.1371/journal.pone.0194670 (PMC5884518; doi:10.1371/journal.pone.0194670)
Supplement: S4 Table — (PDF) [file pone.0194670.s007.pdf]

SUPPORTING INFORMATION

Adaptive differentiation of *Festuca rubra* along a climate gradient revealed by molecular markers and quantitative traits

PLOS One

Bojana Stojanova[*,1,2],Mária Šurinová[1,2], Jaroslav Klápště[3], Veronika Koláriková[1], Věroslava Hadincová[2], Zuzana Münzbergová [1,2]

[1] Department of Botany, Faculty of Science, Charles University, Prague, Czech Republic

[2] Institute of Botany, Academy of Sciences of the Czech Republic, Průhonice, Czech Republic

[3] Scion (New Zealand Forest Research Institute Ltd.), Whakarewarewa, Rotorua, 3046, New Zealand

[*] Corresponding author: bojana.stojanova@gmail.com, tel. +420 271 015 708, Fax +420 271 015 105

S4 Table. Genetic and genotypic diversity of the studied *Festuca rubra* populations.

Estimates were made using SPAGeDi. The sample size is variable and higher than 25 in most populations because we added plants that were sampled in natural populations but not included in the experimental design. All other genetic analyses (QST – FST, COA, Mantel tests, Pons and Petit's index of population genetic diversity of non-ordered alleles) were calculated based solely on the genotypes used in the experiment.

Population	Sample size	Alleles per population	Rarified alelic richness	Genotypic diversity
ALP1	29	33	6.99	0.90
ALP3	32	31	6.07	0.59
ALP4	31	23	4.42	0.16
SUB1	40	44	8.13	0.90
SUB2	58	46	7.31	0.90
SUB3	27	36	7.22	0.67
SUB4	45	39	7.52	0.80
BOR1	30	38	6.71	0.80
BOR2	36	42	7.39	0.97
BOR3	61	43	7.92	0.79
BOR4	37	39	7.14	0.70
